# A prognostic dynamic model applicable to infectious diseases providing easily visualized guides: a case study of COVID-19 in the UK

**DOI:** 10.1038/s41598-021-87882-9

**Published:** 2021-04-16

**Authors:** Yuxuan Zhang, Chen Gong, Dawei Li, Zhi-Wei Wang, Shengda D. Pu, Alex W. Robertson, Hong Yu, John Parrington

**Affiliations:** 1 Department of Pharmacology, University of Oxford, Oxford, OX1 3QT UK; 2 Department of Materials, University of Oxford, Parks Road, Oxford, OX1 3PH UK; 3 Department of Physics, University of California, San Diego, La Jolla, CA USA; 4 Computer Science, University of York, York, YO10 5GH UK; 5 College of Physics, Jilin University, Changchun, 130012 People’s Republic of China; 6 Shanghai Chest Hospital, Shanghai Jiao Tong University, Shanghai, 200030 People’s Republic of China

**Keywords:** Epidemiology, Infectious diseases, Viral infection

## Abstract

A reasonable prediction of infectious diseases’ transmission process under different disease control strategies is an important reference point for policy makers. Here we established a dynamic transmission model via Python and realized comprehensive regulation of disease control measures. We classified government interventions into three categories and introduced three parameters as descriptions for the key points in disease control, these being intraregional growth rate, interregional communication rate, and detection rate of infectors. Our simulation predicts the infection by COVID-19 in the UK would be out of control in 73 days without any interventions; at the same time, herd immunity acquisition will begin from the epicentre. After we introduced government interventions, a single intervention is effective in disease control but at huge expense, while combined interventions would be more efficient, among which, enhancing detection number is crucial in the control strategy for COVID-19. In addition, we calculated requirements for the most effective vaccination strategy based on infection numbers in a real situation. Our model was programmed with iterative algorithms, and visualized via cellular automata; it can be applied to similar epidemics in other regions if the basic parameters are inputted, and is able to synthetically mimic the effect of multiple factors in infectious disease control.

## Introduction

Coronavirus disease (COVID-19) is an infectious respiratory syndrome caused by severe acute respiratory syndrome coronavirus 2 (SARS-CoV-2) and characterised by high overall human-to-human transmission potential^1^. The epidemic rapidly spread regionally at the end of 2019 and subsequent follow on caused a global pandemic and left the world facing a grave social as well as economic crisis^2^. Yet until an effective vaccination programme for all populations or specific medicine to combat COVID-19 is realised, it is likely that the possibility of a resurgence in contagion will exist so that disease control may become a normal part of everyday life^3^. In most world regions, the initial basic reproduction number (R_0_) of COVID-19 was around 2.2–6.47^4–9^. In comparison, for MERS and SARS, the overall R_0_ values were 0.47 and 0.95, respectively^10^, indicating that the transmissibility of COVID-19 is up to 10 times higher than that of previous coronavirus-caused infectious respiratory syndromes. In addition,, the complex epidemiological properties of COVID-19 which include a long infectious period (infectiousness during the incubation period)^11^, the existence of asymptomatic infectors who have a similar infection capacity (30–60%)^10,12^, a high overall self-healing rate (80%)^13^, yet clinical severity in a minority of individuals^14^, have made the disease different from other antecedent epidemics. As such the unprecedentedly enormous infection scale and limited healthcare system capacity makes it challenging to formulate a proper disease control strategy in the COVID-19 era.

Over the last year, many previous epidemic studies have successfully provided insights for understanding, predicting and simulating the development of COVID-19 from multiple perspectives, such as calculating and predicting the evolving basic reproduction number^4^; quantitively predicting epidemiological characteristics^10,15^; evaluating particular disease control measures such as social distancing^16^, controlling mobility^17–20^; analysing transmission dynamics in special populations such as the elderly, obese individuals, and diabetics^3,21,22^; evaluating various factors affecting disease transmission from urban health, meteorological and geo-environmental perspectives, such as water systems, wind speed, and air pollution^23–25^; and also forecasting the subsequent impacts on the social environment and economy^26,27^. Epidemic models evaluating the effect and efficiency of disease control measures have provided good reference points for policy makers^5,17,21,28,29^, and in this study we aim to establish such a model to systematically simulate different measures, and with consideration of the special epidemiological characteristics. Most of the existing models follow the existing principle of susceptible-infectious-recovered (SIR)^7^; here we established a novel infectious-hospitalized-self-heal (IHS) model, with application of an iterative algorithm; asymptomatic infectors, hospitalized infectors, and self-heal infectors were analysed separately. This new principle makes our model more applicable to COVID-19 when considering its huge infection scale and its contagiosity in asymptomatic and pre-symptomatic infectors. With the involvement of some region-specific parameters such as population density, mobility, and hospital capacity, our model is also flexible for application to different global regions where COVID-19 is unlikely to follow an identical path^15^. With the platform of cellular automata, the simulation results are visualized and accessible. In this article, we simulate the development and recovery processes in the UK for 100 days since the first outbreak, and we discuss what is the optimum plan for early-stage disease control, and also the optimal vaccination strategy based on the updated conditions, which will effectively bring the pandemic to an end.

## Results

### Dynamic transmission model

In this article we summarised governmental interventions into three key strategies. First, the intraregional transmission probability has been lowered by protective measures or vaccinations, which aim to reduce the possibility of people contracting the disease^24,30^. Second, the mobility of the population has been reduced by government-level measures like city lock-down, border sealing, and compulsory stay-at-home policies^19^. Last but not least, healthcare system capacity has been enhanced to make sure as many patients as possible are quarantined and treated, while enhancement of detection capacity has aided early detection and immediate isolation^31^. We introduced interregional communication rate (c) to describe the coefficient of disease transmission between communities to take the impact of population mobility into consideration. Initial intraregional growth rate (m) was introduced to describe internal infection among communities, which indicates the influence of personal protection measures such as keeping a social distance and face covering. During the simulated transmission process, intraregional growth rate (m) changes continuously as it is affected by patient recovery and thus gain of immunity. Detection rate of infectors (k) was introduced to describe the possibility of an infector (including asymptomatic and pre-symptomatic individuals) being detected.

$$\begin{aligned} N_{5} \left( {t + 1} \right) & = \left[ {N_{5} \left( t \right) + \left( {N_{2,4,6,8} \left( t \right) - 4N_{5} \left( t \right)} \right) \cdot c} \right] \cdot \left( {1 + m_{5} \left( t \right)} \right) - H_{5} \left( t \right) - S_{5} \left( t \right) \\ H_{5} \left( t \right) & = N_{5} \left( {t - t_{h} } \right) \cdot m_{5} \left( {t - t_{h} } \right) \cdot s \cdot \left( {1 - 4c} \right)^{{t_{h} }} + N_{2,4,6,8} \left( {t - t_{h} } \right) \cdot m_{5} \left( {t - t_{h} } \right) \cdot h \cdot c \cdot \frac{{1 - \left( {1 - 4c} \right)^{{t_{h} }} }}{4c} \\ S_{5} \left( t \right) & = N_{5} \left( {t - t_{s} } \right) \cdot m_{5} \left( {t - t_{s} } \right) \cdot s \cdot \left( {1 - 4c} \right)^{{t_{s} }} + N_{2,4,6,8} \left( {t - t_{s} } \right) \cdot m_{5} \left( {t - t_{s} } \right) \cdot s \cdot c \cdot \frac{{1 - \left( {1 - 4c} \right)^{{t_{s} }} }}{4c} \\ m_{5} \left( {t + 1} \right) & = m \cdot \left( {1 - \frac{1}{{p_{5} }} \cdot \left[ {\mathop \sum \limits_{i = 1}^{t} H_{5} \left( i \right) + \mathop \sum \limits_{i = 1}^{t} S_{5} \left( i \right) + N_{5} \left( t \right)} \right]} \right) \\ \end{aligned}$$

*N*: Daily infection number, *c*: Interregional communication coefficient (travel rate over 15 km^2^), *m*: Self-growth rate (intraregional spreading coefficient), *h*: General percentage of hospitalization (including death), *s*: General percentage of self-healing, *t*_*h*_: Average latent period, *t*_*s*_: Average self-heal period, *H*: Daily hospitalization number, *S*: Daily self-healing number, *p*: Population.

We use cellular automata as a platform of modelling; in cellular automata, cells are arranged as matrixes such as: $$\begin{array}{*{20}c} 1 & 2 & 3 \\ 4 & 5 & 6 \\ 7 & 8 & 9 \\ \end{array} { }$$; each cell represents a region and people tend to migrate between two adjacent cells (details provided in Supplementary Information, Fig. S1). In the equations, N5(t + 1) is the daily infection number on day t + 1 in cell 5, which equals the daily infection number on day t added to the effect of migration in and out, then multiplied by the intraregional spreading coefficient, and subtraction of the day’s number going to hospital and self-healing. The percentage of people who migrate out of a square cell from one side is *c*, therefore, the percentage of people who migrate out of a whole square is 4c.

Then we introduce controlled parameters to describe the situation after interventions (details in “Methods” section). To be realistic, controlled interregional communication rate (c_c_) and detection rate of infectors (k) are steady state values while controlled intraregional growth rate (m_c_) is an initial value varying with the immunity acquisition number.

$$\begin{aligned} N_{5} \left( {t + 1} \right) & = \left\{ {\left[ {N_{5} \left( t \right) + \left( {N_{2,4,6,8} \left( t \right) - 4N_{5} \left( t \right)} \right) \cdot c_{c} } \right] \cdot \left( {1 + m_{5} \left( t \right)} \right) - H_{5} \left( t \right) - S_{5} \left( t \right)} \right\} \cdot \left( {1 - k} \right) \\ H_{5} \left( t \right) & = N_{5} \left( {t - t_{h} } \right) \cdot m_{5} \left( {t - t_{h} } \right) \cdot h \cdot \left( {1 - c_{c} } \right)^{{t_{h} }} + N_{2,4,6,8} \left( {t - t_{h} } \right) \cdot m_{5} \left( {t - t_{h} } \right) \cdot h \cdot c_{c} \cdot \frac{{1 - \left( {1 - c_{c} } \right)^{{t_{h} }} }}{{c_{c} }} \\ S_{5} \left( t \right) & = N_{5} \left( {t - t_{s} } \right) \cdot m_{5} \left( {t - t_{s} } \right) \cdot s \cdot \left( {1 - c_{c} } \right)^{{t_{s} }} + N_{2,4,6,8} \left( {t - t_{s} } \right) \cdot m_{5} \left( {t - t_{s} } \right) \cdot s \cdot c_{c} \cdot \frac{{1 - \left( {1 - c_{c} } \right)^{{t_{s} }} }}{{c_{c} }} \\ m_{5} \left( {t + 1} \right) & = min\left\{ {m \cdot \left( {1 - \frac{1}{{p_{5} }} \cdot \left[ {\mathop \sum \limits_{i = 1}^{t} H_{5} \left( i \right) + \mathop \sum \limits_{i = 1}^{t} S_{5} \left( i \right) + N_{5} \left( t \right)} \right]} \right),m_{c} } \right\} \\ \end{aligned}$$

*m*_*c*_: Initial value of controlled intraregional growth rate, *c*_*c*_: Controlled interregional communication rate, *k*: Detection rate of infectors, *p*: Regional population.

We also take advantage of the definition of m which initially relies on R_0_ and changes with the percentage of immunization to calculate the number of vaccinations required in disease control.

$$m_{c} = m \cdot \left( {1 - \frac{{v_{r} + v + i}}{p}} \right)$$

*v*_*r*_: Required vaccinations, *v*: Received vaccinations, *i*: Cumulative number of infections.

### Visualised transmission process of COVID-19 in the UK

**Figure 1:**
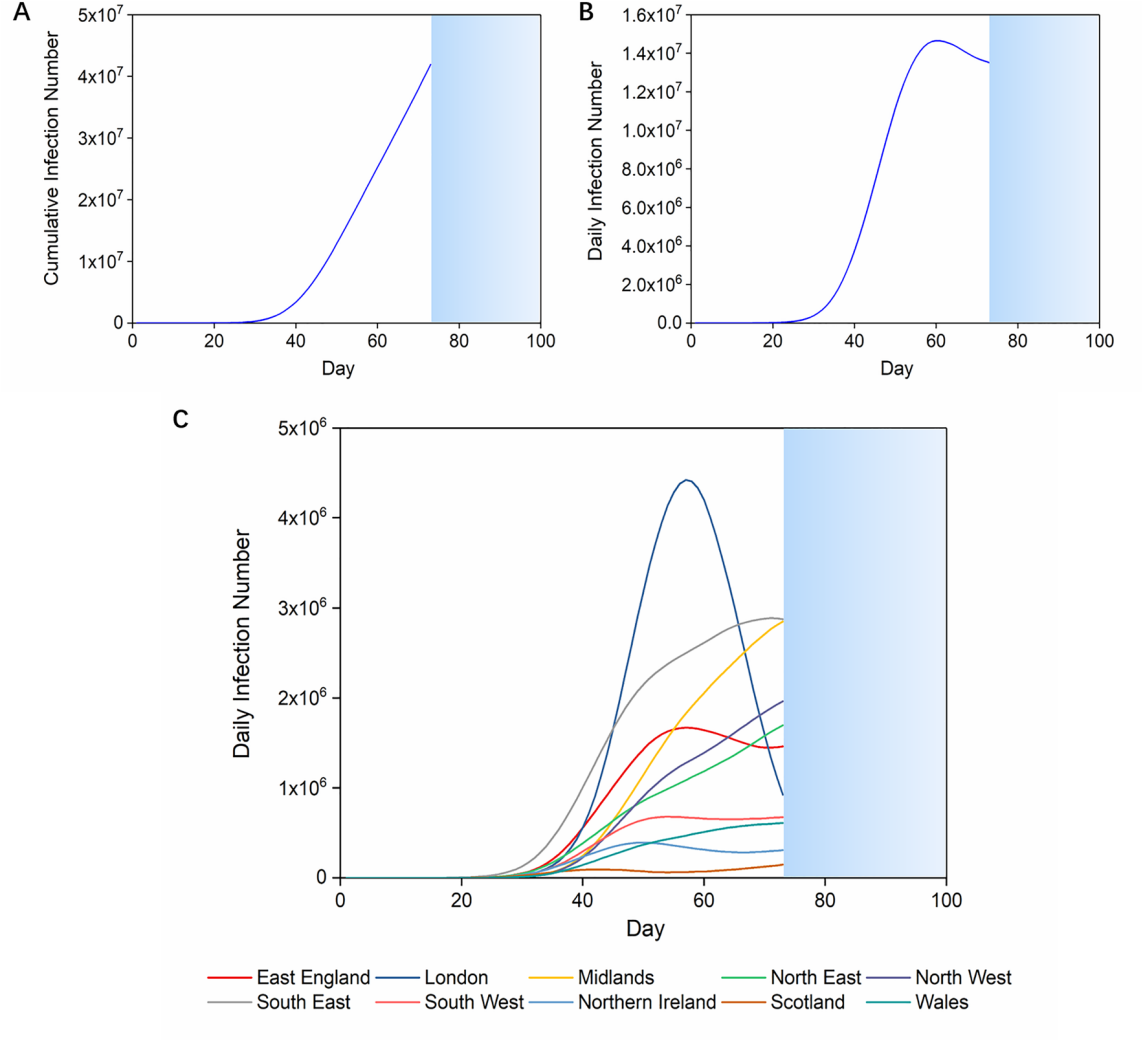
Infection curves without interventions in the UK. (**A**,**B**) The cumulative/daily infection curve without interventions in the UK. The simulation stops when cumulative infection number exceeds the regional population (blue sections). (**C**) The daily infection curves for regions in the UK without interventions. The simulation stops when cumulative infection number exceeds the regional population.

**Figure 2:**
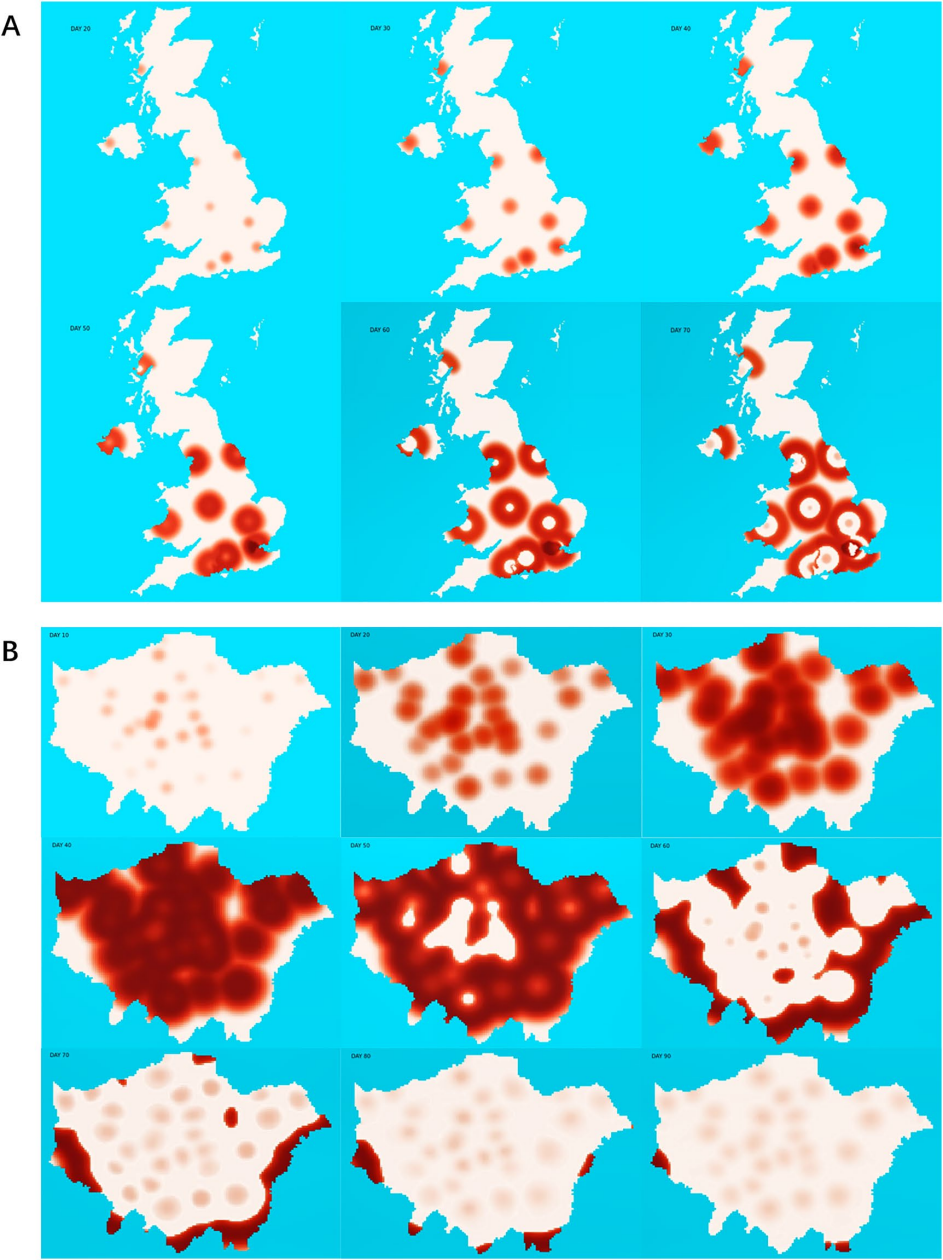
Visualized COVID-19 transmission without interventions in the UK and London. (**A**) Screenshots of visualized dynamic model of transmission in the UK from day 10 to day 60. The red saturation represents the severity of COVID-19. COVID-19 starts from a random spot in each region and spreads rapidly. In the outbreak centre, COVID-19 infection will reach a peak on day 50 and be cleared on day 60 when it is out of control in the whole country. (**B**) Screenshots of visualized dynamic model of transmission in London from day 10 to day 90. (Day-by-day transmission videos were generated by Pythons 3 https://www.python.org using python package matplotlib https://matplotlib.org with initial maps from open-source software WordPress.org. Screenshots were generated by Windows10, more details were shown in the Results——Technical details.)

Videos showing the prognostic transmission process on the UK and London maps were generated by Python 3 based on our model (details shown in “Methods” section and Supplementary Information). Here we present the infection curves (Fig. 1) and visualised transmission maps (Fig. 2) based on our simulation of the infection which starts with the initial daily infection number of each region in the UK on 4th March 2020^32^. Due to the limitations of the iterative algorithm, the earliest date we can start simulating disease control interventions is one day after the first self-heal period. Therefore, in the first 15 days (a self-heal period) of the simulation, we use the initial parameters according to COVID-19 epidemiological characteristics, the initial intraregional growth rate as 0.4^8,33^, the interregional travel rate as 0.1^38^, and the detection rate of infectors as 0 (Table 1, data resources presented in “Methods” section). Without any governmental intervention, the accumulated infection number in 100 days may exceed 70% of the UK population (Fig. 1A). Since the simulation was interrupted when the cumulative infection number exceeds the local population, COVID-19 transmission in the UK suspends on the 73rd day (Fig. 1A). The daily infection scale will exceed 14.8 million people on the 60th day of domestic infection (Fig. 1B), which accounts for nearly a quarter of the UK population^34^. When we look at the regional infection curves, we can see that the epidemic in London develops differently from other regions in that the infection in the former increases sharply from the 30th to the 58th day, then decreases rapidly (Fig. 1C). This indicates that, without interventions, the infection will first be eliminated in London while the conditions are still getting worse in other regions.

**Table 1:** Parameter symbols, definitions and values used as initial conditions in this study.

Symbol	Definitions	Value	References
R_0_	Basic reproduction number	2.2–6.94	^8,33^
m	Initial spreading coefficient	0.4	^8^
c	Interregional communication coefficient	0.1 (UK)/0.2 (London)	^38,42^
h	Percentage of hospitalization including death rate	0.2	^49,50^
s	Percentage of self-healing	0.8	^32^
t_h_	Average incubation period	6	^18^
t_s_	Average self-heal period	15	^46^
v	Received vaccinations (until Jan 2021)	462,114	^32^
i	Cumulative number of infections (until Jan 2021)	3,817,176 (UK)/652,979 (London)	^32^
R_0-D614G_	Basic reproduction number of variant D614G	3.1–4.8	^40^

From the visualized transmission maps we can also see that when the infection peak is reached, the COVID-19 outbreak will ameliorate in the epicentre while it is still developing in surrounding regions (Fig. 2A). As shown in the screenshots of the simulation videos (Supplementary video files), the epidemic spreads from the epicentre to the periphery and surprisingly leaves the centre epiclean (Fig. 2A). Here we also simulated the COVID-19 transmission process in London based on the initial daily infection number of each borough on 11th March (Fig. 2B) which shows similar results^32^. Despite the fact that a second wave of infection may occur from the epicentre of the outbreak once again, the periphery may be the place hit by the epidemic more severely in a later period.

### COVID-19 can be brought under control by a single intervention at the early stage, but at huge expense

**Figure 3:**
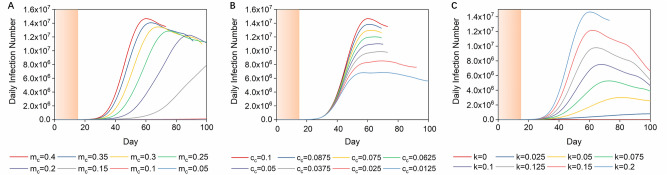
Impact of single interventions on daily infection curves. (**A**) Daily infection curves for the UK with intraregional growth rates varying from 0.4 to 0.05. The orange section means no interventions were taken. (**B**) Daily infection curves for the UK with interregional communication rates varying from 0.1 to 0.0125. (**C**) Daily infection curves for the UK with detection rates of infectors varying from 0 to 0.2.

We ran simulations to see how effective single interventions are in flattening the daily infection curves. When the controlled intraregional growth rate (m_c_) was in the range of 0.05–0.4 (0.75 < R_0_ < 6, $m = R_{0} /15\,{\text{days}}$, details in the “Methods” section)^14,35^, the daily infection curve became progressively flatter with reduced m_c_ while the period of the epidemic became longer. When the controlled intraregional growth rate was lower than 0.1 (R_0_ < 1.5), this was effective in controlling the propagation tendency, but a second infection wave was likely to occur. In addition, it was not possible to completely eliminate infection cases within a 100-day period by only controlling m_c_, and the epidemic would thus last for a longer time (Fig. 3A). When the controlled travel parameter (c_c_) was in the range of 0.0125–0.01, the overall infection trends were downward but it was not possible to control the daily infection scale to an acceptable level (Fig. 3B). An increasing detection rate of infectors (k) was capable of controlling the infection scales stably and efficiently as well as eliminating the infection within a short period (Fig. 3C). The daily infection number dropped while k was enhanced, and as shown in Fig. 3, controlling k brings better disease control results than controlling m_c_ and c_c_. When k is as high as 0.175, it was possible to maintain the daily infection number curve at a flat level.

### Combined interventions will significantly enhance disease control efficiency

**Figure 4:**
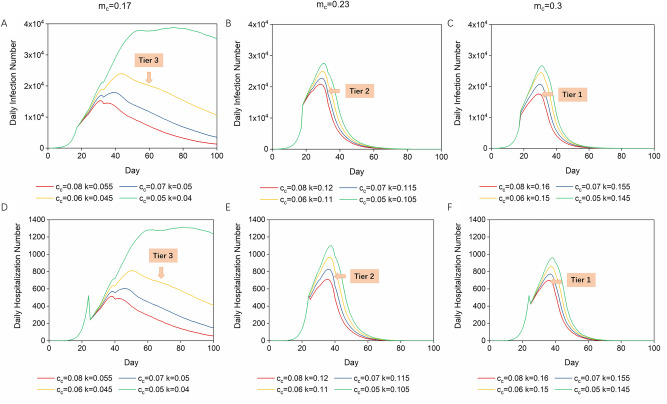
Daily infection curve and daily hospitalization curve with combined interventions. m_c_ was in the range of 0.3–0.17 (initial R_0_ in the range of 4.5–2.5), and c_c_ was in the range 0.05–0.08 (20–32% people in the UK can travel over 2.5 miles a day). (**A**) Daily infection curves when m_c_ was initially controlled at 0.17 (R_0_ = 2.5). (**B**) Daily infection curves when m_c_ was initially controlled at 0.23 (R_0_ = 3.5). (**C**) Daily infection curves when m_c_ was initially controlled at 0.3 (R_0_ = 4.5). (**D**) When m_c_ = 0.17, c_c_ = 0.08–0.05, the number to hospital can be controlled at around 1000 when k is controlled at 0.055–0.045. (**E**) When m_c_ = 0.23, c_c_ = 0.08–0.05, the number to hospital can be controlled at around 1000 when k is controlled at 0.105–0.12. (**F**) When m_c_ = 0.3, c_c_ = 0.08–0.05, the number to hospital can be controlled around 1000 when k is controlled at 0.16–0.145.

As shown in Fig. 3, with achievable single interventions, it is hard to contain the peak daily infection number to acceptable levels. Therefore, as shown in Fig. 4, combined interventions were applied to search for optimum disease control strategies.

The average length of the hospital stay for COVID-19 patients is 7 days^36^. The number of inpatient beds available for COVID-19 patients at the early stage of COVID-19 in the UK was around 6000–7000^32^. So the daily number of hospitalizations should be kept at around 1,000, which is a premise for our optimum disease control strategies. As shown in mobility trend reports by Apple Maps, the general mobility in the UK was reduced by 20–50% during the COVID-19 lockdowns (January 2020–January 2021)^37^. Hence, we suppose the interregional communication rate in the UK to be reduced from 0.1 to 0.08–0.05^38^, indicating that 20% ~ 32% people can travel beyond 4 km (2.5 miles) every day. We selected several representative conditions to quantify the UK tier system, according to this tier system being based on the mobility trends and four-tier alert levels: m_c_ = 0.17 (R_0_ = 2.5), m_c_ = 0.23 (R_0_ = 3.5) and m_c_ = 0.3 (R_0_ = 4.5) with c_c_ in the range of 0.08–0.05 (Table 2)^32,35,37^; then we ran simulations and found proper relevant detection rates. The disease control process occurring systematically with three interventions was simulated and presented as transmission curves (Fig. 4a, b, c). With regard to these different combinations of interventions, we can conclude that when tier 1, tier 2, and tier 3 lockdown measures are implemented, the detection rate should be ensured as 16%, 11.5% and 4.5%, and as shown in Fig. 4d, e, f, the highest daily numbers to hospital (H) under these conditions do not exceed hospital capacity.

**Table 2:** The detection number and vaccination demand under different intervention levels.

Restriction level	Controlled R_0_	Controlled intraregional growth rate (m_c_)	Controlled interregional communication rate (c_c_)	Detection rate (k)	Vaccinations (million)
**London**					
Tier 4	1.5	0.1	0.05	0.05	4.74
Tier 3	2.5	0.17	0.06	0.08	3.15
Tier 2	3.5	0.23	0.07	0.11	2.21
Tier 1	4.5	0.3	0.08	0.15	0.7
**UK**					
Tier 4	1.5	0.1	0.05	0.05	42.73
Tier 3	2.5	0.17	0.06	0.09	32.85
Tier 2	3.5	0.23	0.07	0.13	28.18
Tier 1	4.5	0.3	0.08	0.2	19.9

### Application for calculating vaccination demand to end COVID-19

**Figure 5:**
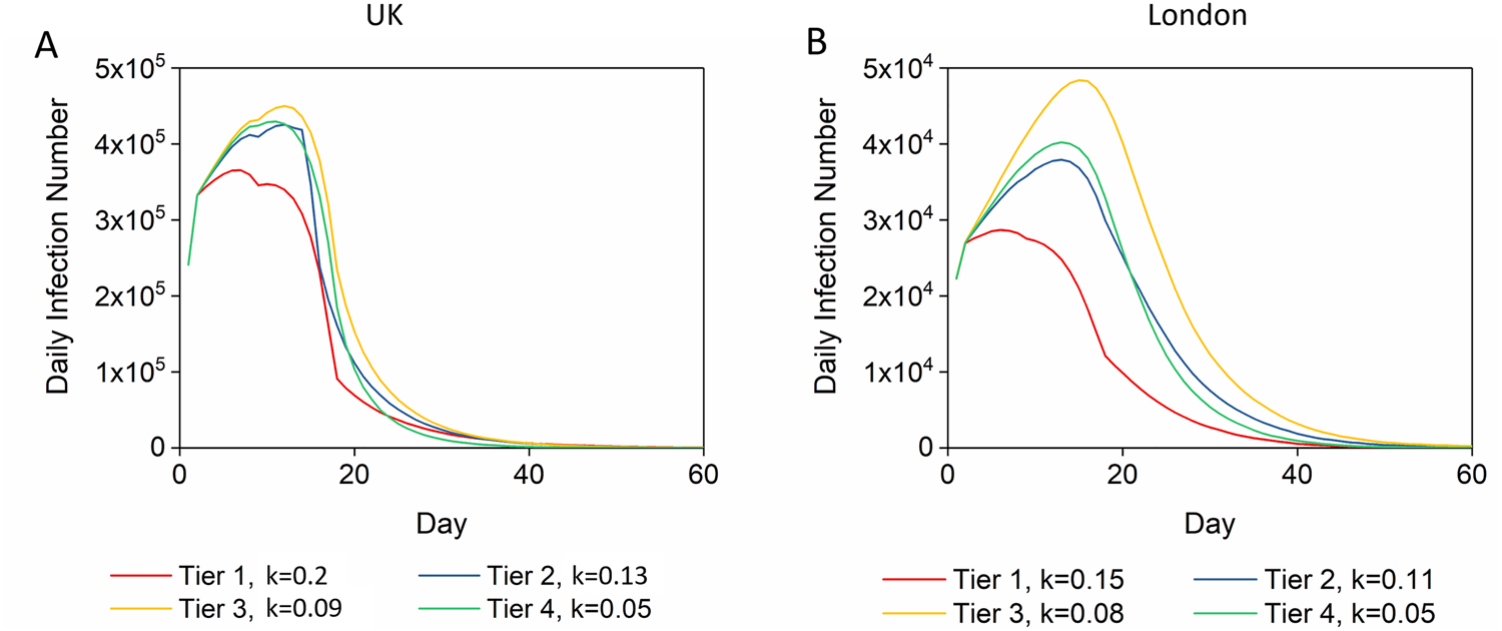
The daily infection curves at the recovery stage with proper control strategies. (**A**,**B**) Ideal daily infection curves starting with a daily infection number of 220,000 in the UK/22,000 in London.

A key current strategy to combat COVID-19 is the development of an effective vaccine. We thereby provided a method of calculating the required vaccination numbers based on this model. We started the simulation with the current infection number as an initial condition, and intraregional growth rate was influenced by vaccination and existing natural immunity. For example, in early January 2021, the average daily infection cases in the UK were around 35,000 with 3,300 being in London^39^. Considering the average infection period of COVID-19 which is 2–15 days^10^, we assume the real infection number in the UK and London to be 220,000 and 22,000^32^, accordingly. With the premise of controlling the pandemic within two months, and controlling m_c_ solely with vaccinations, we simulated the disease control process with the initial infection number of 220,000 and 22,000 in the UK and London and calculated the required number of vaccinations based on optimized m_c_ values (Fig. 5) by applying the equation of $m_{c} = m \cdot \left( {1 - \frac{{v_{r} + v + i}}{p}} \right)$ as presented in an earlier paragraph^32^. The required number of vaccinations should be not less than 43 million in the UK and 4.2 million in London (Table 2).

## Discussion

As we have summarised, intervention measures have focused on three aspects: the intraregional spreading rate, detection rate of infectors and interregional communication rate. Long-duration intraregional lockdown effectively reduced the burden of the pandemic^19,20^, however, without cutting off the source of infection, the epidemic will not be eliminated in 100 days, even if the R_0_ number is very low (Fig. 3A). With respect to interregional communication rate, during the first round lockdown and associated impact on travel, 46% of driving, 62% of public transport and 33% walking trips, were reduced on the days of lock-down compared to normal days^37^. However, the data shows the reduction started from 21st March^37^, when the cases of infection had already spread all over the country, therefore intraregional growth was already occurring at this point^35^. This also matches our simulation results and shows that it is difficult to control the infectious trend by simply reducing the travel parameter once the infection has spread to all regions. We then considered rate of detection and quarantine for infectors. In our simulation, around one fifth of infectors must be detected and strictly isolated even if they are in the latent period of the disease or asymptomatic, and enhancing detection rate of infectors (k) is shown to be the most efficient intervention to bring the infection scale down as well as shorten the intervention period.

However, controlling all the single disease control parameters to ideal values is difficult in real conditions because of the special characteristics of COVID-19. Detection and isolation of early-stage and asymptomatic infectors is a big challenge for healthcare systems, and this was particularly the case with the immature detection technologies and limited resources in the first phase of the COVID-19 outbreak^14,15^. Therefore, our findings support the conclusion that COVID-19 spread must be controlled by multiple combined strategies and as early as possible (Supplementary Information Fig. S2). The initial R_0_ value was around 5.81 in the UK^9^. To reduce the social burden as well as balance the needs of the economy and disease control, we believe that controlling R_0_ within the range of 2.5–4.5 and mobility reduced by 20–32% (tier 1–3) is a reachable goal with proper control measures taken at the beginning of the period of interventions^17,32,35^. To keep the peak daily number of hospitalizations within acceptable levels when R_0_ is immediately controlled at 2.5 (tier 3), with intermediate travelling control policy, 5% of infectors must be detected and quarantined. When R_0_ is controlled at 4.5 (tier 1), our recommendation is that the detection rate should be enhanced to at least 20%.

Moreover, our simulation showed that the location at which the epidemic is most severe, was where the epidemic first began to disappear. So we suggest that instead of gathering all detection systems and resources to the main areas affected by the epidemic, distributing these resources to the peripheral regions will be a more efficient way to save resources and bring the epidemic under control. We also provided a potential method of calculating required vaccination numbers based on the actual infection number, for example, our simulation shows that when the infection number is around 220,000 in the UK and 22,000 in London^32^, the number of vaccinations in the UK should not be less than 45 million in the UK and 6.6 million in London.

Our study presents a few limitations due to model design as well as the nature of cellular automata. One such limitation is the inability of cellular automata to mimic long-distance migrations like trips by plane during the early stage of the disease transmission, as the chroma are only transmitted between two adjacent cells at a time (Supplementary Information Fig. S1). Another limitation is that mimicking the change of R_0_ through the process of viral mutation is not applicable. Future optimizations of such modelling studies may focus on plugging evolving parameters relating to the variations in the virus in the longer term^40^. It will also be interesting to introduce new parameters to quantify other critical factors affecting epidemic transmission from social, economic, environmental, demographic, climatological, and health risk angles^23,25,26,41^.

## Conclusion

This study is a prognostic analysis of infectious disease development on the strength of an infectious-hospitalized-self-heal (IHS) mathematic model with the first wave of COVID-19 in the UK as an example. The model is designed to match the epidemiological characteristics of infectious diseases with similarities to COVID-19, in particular ones with asymptomatic and pre-symptomatic infectivity.

Through Python design, we realized the systematic regulation of intraregional growth rate, interregional communication coefficient, and detection rate. It is easy to evaluate the disease control effect by adjusting parameters and thus we can seek optimal solutions. In addition, we have found that to achieve better control effects in the mid-term of the epidemic, more attention should be paid to the surrounding areas of the epicentre. Moreover, our model can also be applied to estimate the quantity of vaccination demand based on realistic situations to provide guidance for vaccination production.

This model can also be applied in the future to predict the spread of similar infectious diseases in different regions. It only needs to input specific disease parameters in the system, such as incubation period, self-healing period, self-healing rate and so on. This model makes it convenient to quickly find the optimal solutions for comprehensive interventions and take action, which can be helpful in future public health decision-making to reduce morbidity and mortality.

## Methods

### Assumptions

1. The population is approximated to be constant and evenly distributed within each geographical region.
2. Death rate is counted as a part of the percentage of cases that are admitted to hospital.
3. Infected people are contagious constantly from the beginning to the end of the incubation period as well as during the illness stage.
4. All the population are at the same risk of infection.
5. All patients have contracted the virus through secondary infection; considering the high population mobility, primary infected patients, which represent a tiny percentage, are omitted.

### Automata cell establishment

Cellular automata is a dynamic system that is discrete in time and space; it consists of a regular grid of cells, with each one being in a finite number of states. In our model, the disease transmission was described as partial cellular interaction leading to global change. A geographical region was regarded as a two-dimension network. To input this into cellular automata, each network was deemed as a cell while each cell stands for the location of a group of people. Pixels were downscaled to correspond to the area of the cells. Each cell was selected and separated according to the red, green, blue (RGB) value of the map (Supplementary Information Fig. S1). Red colour chromatic value in the pixels represents the severity of the epidemic in the corresponding regions. The minimum value (r = 0) means no cases while the maximum value (re = 225) represents the population of the cell.

The epidemic information and regional population of each cell was set initially^32,34^, and the number of people who migrate each day depends on the interregional communication coefficient and the local population.

A method of convolution kernel was applied for calculation of migrating cases. We suppose that infection starts from one cell in each region, which was represented as a red dot on the map. The location of the dot was randomly chosen, and pseudo-random number seeds were fixed. The epidemic is assumed not to transmit to non-populated areas outside the coast where the infection number was forcibly set as zero.

### Dynamical equations

Infectors go to the hospital and become self-healed only after the appearance of symptoms, and thus the precise number that go to hospital and self-heal depends on the infection number one period previously. The day’s number to hospital in the cell is dependent on the daily infection number that occurred six days (the average latent period) previously in local and surrounding regions. Considering that some infectors will continually migrate between regions, these infectors who are infected 6 days previously and are currently in cell 5 can be divided into two parts: infectors who were infected in cell 5 and remained in cell 5 (local infectors who never migrate), and infectors who were infected in other cells and moved into the cell 5 in the previous 6 days. Assuming the number of infectors in cell 5 at the beginning is Y_5_.

$$\begin{array}{*{20}l} {{\text{Day}}} \hfill & {\text{Number of local infectors in cell 5}} \hfill \\ 1 \hfill & {Y_{5} \left( {1 - 4c} \right)} \hfill \\ 2 \hfill & {Y_{5} \left( {1 - 4c} \right)^{2} } \hfill \\ 3 \hfill & {Y_{5} \left( {1 - 4c} \right)^{3} } \hfill \\ \cdot \hfill & {} \hfill \\ \cdot \hfill & {} \hfill \\ T \hfill & {Y_{5} \left( {1 - 4c} \right)^{t} } \hfill \\ \end{array}$$

Therefore, replacing Y with the exact number of infectors, the daily number to hospital of local infectors in cell 5 on day t is calculated as

$$N_{5} \left( {t - t_{h} } \right) \cdot m_{5} \left( {t - t_{h} } \right) \cdot h \cdot \left( {1 - 4c} \right)^{{t_{h} }}$$

Next, we consider the number of infectors who move into cell *5*. Suppose the number of people in group *Y* in adjacent cells of *Y*_*5*_ are *Y*_*2*_*, Y*_*4*_*, Y*_*6*_*, Y*_*8*_, and add up to *Y*_*2,4,6,8*_. Since each cell has only one side in contact with cell *5*, on the first day the number of people in group *Y* who move into cell *5* is $Y_{2,4,6,8} \cdot c$. Meanwhile people also move out from cell *5*, so on the second day the number of people in group *Y* who move into cell *5* is $Y_{2,4,6,8} \cdot c \cdot \left( {1 - 4c} \right)$. The rest can be calculated in the same manner.

$$\begin{array}{*{20}l} {{\text{Day}}} \hfill & {{\text{Number of people in group}}\,Y\,{\text{who move into cell}}\,5} \hfill \\ 1 \hfill & {Y_{2,4,6,8} \cdot c} \hfill \\ 2 \hfill & {Y_{2,4,6,8} \cdot c\left( {1 - 4c} \right)} \hfill \\ 3 \hfill & {Y_{2,4,6,8} \cdot c\left( {1 - 4c} \right)^{2} } \hfill \\ \cdot \hfill & {} \hfill \\ \cdot \hfill & {} \hfill \\ t \hfill & {Y_{2,4,6,8} \cdot c\left( {1 - 4c} \right)^{t - 1} } \hfill \\ \end{array}$$

Hence, the total number of people in group *Y* who move into cell *5* on day *t* is

$$\mathop \sum \limits_{i = 0}^{t - 1} Y_{2,4,6,8} \cdot c\left( {1 - 4c} \right)^{i} = Y_{2,4,6,8} \cdot c \cdot \frac{{1 - \left( {1 - 4c} \right)^{t} }}{{1 - \left( {1 - 4c} \right)}} = Y_{2,4,6,8} \cdot c \cdot \frac{{1 - \left( {1 - 4c} \right)^{t} }}{4c}$$

If we replace *Y* with the exact number of infectors, the daily number of infectors who move into hospital in cell 5 on day t is calculated as

$$N_{2,4,6,8} \left( {t - t_{h} } \right) \cdot m_{5} \left( {t - t_{h} } \right) \cdot h \cdot c \cdot \frac{{1 - \left( {1 - 4c} \right)^{t} }}{4c}$$

Therefore, the daily increase in the number of hospitalizations in cell *5* on day *t* is

$$H_{5} \left( t \right) = N_{5} \left( {t - t_{h} } \right) \cdot m_{5} \left( {t - t_{h} } \right) \cdot s \cdot \left( {1 - 4c} \right)^{{t_{h} }} + N_{2,4,6,8} \left( {t - t_{h} } \right) \cdot m_{5} \left( {t - t_{h} } \right) \cdot h \cdot c \cdot \frac{{1 - \left( {1 - 4c} \right)^{{t_{h} }} }}{4c}$$

In a similar way, the daily increase in the number that self-heal (S) is calculated as

$$S_{5} \left( t \right) = N_{5} \left( {t - t_{s} } \right) \cdot m_{5} \left( {t - t_{s} } \right) \cdot s \cdot \left( {1 - 4c} \right)^{{t_{s} }} + N_{2,4,6,8} \left( {t - t_{s} } \right) \cdot m_{5} \left( {t - t_{s} } \right) \cdot s \cdot c \cdot \frac{{1 - \left( {1 - 4c} \right)^{{t_{s} }} }}{4c}$$

### Data sources

We used an initial spreading coefficient to explain the daily percentage increase; in the UK the initial R_0_ value was 5.81^8,9^, the infection period (including incubation period) was 15 days, and the incidence number doubled every 1.8–2.8 days (Table 1). So the value of the initial growth rate can be calculated as 0.4 $\left( {m = R_{0} /15\,{\text{days}}} \right)$.

We set 16,183 pixels, for the areas of the UK. Therefore, on the UK map, each pixel represents a 15 km^2^ geographic area, and people who travel over a 4 km (2.5 mile) straight-line distance are considered as migrants. The percentage of people migrating between cells in the UK is around 40% as roughly estimated based on available worldwide and domestic travel and transport statistics (Table 1)^38,42^. Since there are four directions in which people in one square cell can migrate, the number will be divided by 4, and the travel parameter was estimated to be 0.1, standing for 10% people in one cell migrating between two adjacent cells every day (Table 1).

The general percentage of hospitalization means the possibility of infectors being accepted to the hospital and thus strictly isolated. We consulted the cumulative death rate which was estimated at 15.4%, and the number of beds occupied by confirmed COVID-19 patients according to the NHS statistics in July, which showed that 2000 beds were occupied by COVID-19 patients^43^. Moreover, in early April the number of hospitalizations was estimated at around 7000–8000^44^. Considering there to be 200% undetected cases, as the number of undetected patients is estimated to be more than two times that of the confirmed patients, the percentage to hospital including death rate is 20% (Table 1)^45^.

Since the illness period is estimated at 15 days, the controlled spreading coefficient can be calculated as $m = R_{0} /15\,{\text{days}}$, which means the average number of people who can contract COVID-19 from one patient in one day during his/her illness period^46^.

The detection rate of infectors stands for the possibility of an infector being detected as well as isolated. For instance, if the healthcare system provides no detection service, the detection rate of infectors is equal to 0. If the healthcare system provides enough detection for all patients with severe symptoms and immediately isolates them, the detection rate of infectors is equal to the rate of occurrence of severe symptoms (13.8%)^46^. If the healthcare system provides general, extensive and compulsive detection services for all citizens, the detection rate of infectors will be close to 1.

An approximate validation of the accuracy of the model was based on the early statistics from the UK government, although this was hard to do in practice because the real transmission dynamic and infection scale were difficult to determine at the early stage of the pandemic (Supplementary Table S1).

### Technical details

All source codes were generated by Python 3 (https://www.python.org) and Jupyter Notebook (jupyter.org). Videos were produced by python package matplotlib (https://matplotlib.org) using "Animation" Class. Initial UK and London maps were downloaded from open-source software WordPress.org which was released under a General Public License (GPLv2) from the Free Software Foundation^47,48^, and were pre-processed by python package scikit-image (https://scikit-image.org) so that all boundaries between regions/boroughs are smoother for mimicking population migration. All codes are publicly available on GitHub (https://github.com/daweiliucsd/Cov19-model). In order to run all example codes, prebuilt Python distributions such as Anaconda are strongly recommended.

## Supplementary information

Supplementary information 1.

Supplementary Video 1.

Supplementary Video 2.

Supplementary Video 3.

Supplementary Video 4.

Supplementary Video 5.

Supplementary Video 6.

Supplementary Video 7.

Supplementary Video 8.

Supplementary Video 9.

Supplementary Video 10.

Supplementary Video 11.

Supplementary Video 12.

Supplementary Video 13.

## References

[1] WHO. Report of the WHO-China Joint Mission on Coronavirus Disease 2019 (COVID-19). (2020).

[2] WHO. Archived: WHO Timeline—COVID-19. https://www.who.int/news-room/detail/27-04-2020-who-timeline---covid-19.

[3] Kissler, S. M., Tedijanto, C., Goldstein, E., Grad, Y. H. & Lipsitch, M. Projecting the transmission dynamics of SARS-CoV-2 through the postpandemic period. *Science* (2020). 10.1126/science.abb5793. 10.1126/science.abb5793 PMC7164482 32291278

[4] Chintalapudi N, Battineni G, Sagaro GG, Amenta F (2020). COVID-19 outbreak reproduction number estimations and forecasting in Marche, Italy. Int. J. Infect. Dis..

[5] Koo JR (2020). Interventions to mitigate early spread of SARS-CoV-2 in Singapore: a modelling study. Lancet. Infect. Dis.

[6] Wu JT, Leung K, Leung GM (2020). Nowcasting and forecasting the potential domestic and international spread of the 2019-nCoV outbreak originating in Wuhan, China: a modelling study. Lancet.

[7] Gatto M (2020). Spread and dynamics of the COVID-19 epidemic in Italy: effects of emergency containment measures. Proc Natl Acad Sci USA.

[8] Sanche S (2020). High contagiousness and rapid spread of severe acute respiratory syndrome coronavirus 2. Emerg. Infect. Dis..

[9] Dropkin G (2020). COVID-19 UK lockdown forecasts and R0. Front. Public Health.

[10] Cevik M (2021). SARS-CoV-2, SARS-CoV, and MERS-CoV viral load dynamics, duration of viral shedding, and infectiousness: a systematic review and meta-analysis. Lancet Microbe.

[11] Linton NM (2020). Incubation period and other epidemiological characteristics of 2019 novel coronavirus infections with right truncation: a statistical analysis of publicly available case data. JCM.

[12] Kwok KO, Lai F, Wei WI, Wong SYS, Tang JWT (2020). Herd immunity—estimating the level required to halt the COVID-19 epidemics in affected countries. J. Infect..

[13] Wilder-Smith A, Chiew CJ, Lee VJ (2020). Can we contain the COVID-19 outbreak with the same measures as for SARS?. Lancet. Infect. Dis.

[14] Stawicki S (2020). The 2019–2020 novel coronavirus (severe acute respiratory syndrome coronavirus 2) pandemic: a joint american college of academic international medicine-world academic council of emergency medicine multidisciplinary COVID-19 working group consensus paper. J Global Infect Dis.

[15] Jewell NP, Lewnard JA, Jewell BL (2020). Predictive mathematical models of the COVID-19 pandemic: underlying principles and value of projections. JAMA.

[16] Farboodi, M., Jarosch, G. & Shimer, R. *Internal and External Effects of Social Distancing in a Pandemic*. w27059 http://www.nber.org/papers/w27059.pdf (2020). 10.3386/w27059.

[17] Li Y (2021). The impact of policy measures on human mobility, COVID-19 cases, and mortality in the US: a spatiotemporal perspective. IJERPH.

[18] Chang S (2021). Mobility network models of COVID-19 explain inequities and inform reopening. Nature.

[19] Imperial College COVID-19 Response Team *et al.* Estimating the effects of non-pharmaceutical interventions on COVID-19 in Europe. *Nature* **584**, 257–261 (2020). 10.1038/s41586-020-2405-7 32512579

[20] Islam, N. *et al.* Physical distancing interventions and incidence of coronavirus disease 2019: natural experiment in 149 countries. *BMJ* m2743 (2020). 10.1136/bmj.m2743. 10.1136/bmj.m2743 PMC7360923 32669358

[21] Kucharski AJ (2020). Early dynamics of transmission and control of COVID-19: a mathematical modelling study. Lancet. Infect. Dis.

[22] CMMID COVID-19 working group *et al.* Age-dependent effects in the transmission and control of COVID-19 epidemics. *Nat Med* (2020). 10.1038/s41591-020-0962-9. 10.1038/s41591-020-0962-9 32546824

[23] Coccia M (2020). How do low wind speeds and high levels of air pollution support the spread of COVID-19?. Atmos Pollut Res.

[24] Gormley M, Aspray TJ, Kelly DA (2020). COVID-19: mitigating transmission via wastewater plumbing systems. Lancet Glob. Health.

[25] Coccia M (2020). An index to quantify environmental risk of exposure to future epidemics of the COVID-19 and similar viral agents: Theory and practice. Environ. Res..

[26] Yang, C. *et al.* Taking the pulse of COVID-19: a spatiotemporal perspective. *Null* **13**, 1186–1211 (2020).

[27] Wolf M. The risks of lifting lockdowns prematurely are very large. *Financial Times*.

[28] Kraemer MUG (2020). The effect of human mobility and control measures on the COVID-19 epidemic in China. Science.

[29] Prem K (2020). The effect of control strategies to reduce social mixing on outcomes of the COVID-19 epidemic in Wuhan, China: a modelling study. Lancet Public Health.

[30] Greenhalgh, T., Schmid, M. B., Czypionka, T., Bassler, D. & Gruer, L. Face masks for the public during the covid-19 crisis. *BMJ* m1435 (2020). 10.1136/bmj.m1435. 10.1136/bmj.m1435 32273267

[31] Peck, K. R. Early diagnosis and rapid isolation: response to COVID-19 outbreak in Korea. *Clinical Microbiology and Infection* S1198743X20302330 (2020). 10.1016/j.cmi.2020.04.025. 10.1016/j.cmi.2020.04.025 PMC7182747 32344168

[32] https://coronavirus.data.gov.uk/. Coronavirus (COVID-19) in the UK.

[33] Tang B (2020). Estimation of the transmission risk of 2019-nCov and its implication for public health interventions. SSRN J..

[34] *Population of England in 2019, by region*. https://www.statista.com/statistics/294681/population-england-united-kingdom-uk-regional/.

[35] Hale, T., Petherick, A., Phillips, T. & Webster, S. Variation in government responses to COVID-19. *Version 2.0. Blavatnik School of Government Working Paper*.

[36] Rees, E. M. *et al. COVID-19 length of hospital stay: a systematic review and data synthesis*. 10.1101/2020.04.30.20084780 (2020). 10.1101/2020.04.30.20084780. 10.1186/s12916-020-01726-3 PMC7467845 32878619

[37] APPLE MAPS. Mobility Trends Reports. https://www.apple.com/covid19/mobility.

[38] UK.GOV. Transport Statistics Great Britain: 2019. https://www.gov.uk/government/statistics/transport-statistics-great-britain-2019.

[39] Coronavirus (COVID-19) Cases. https://data.london.gov.uk/dataset/coronavirus--covid-19--cases.

[40] Volz E (2021). Evaluating the effects of SARS-CoV-2 spike mutation D614G on transmissibility and pathogenicity. Cell.

[41] Coccia M (2020). Factors determining the diffusion of COVID-19 and suggested strategy to prevent future accelerated viral infectivity similar to COVID. Sci. Total Environ..

[42] London Datastore. Coronavirus (COVID-19) Mobility Report. https://data.london.gov.uk/dataset/coronavirus-covid-19-mobility-report.

[43] NHS. Bed Availability and Occupancy. https://www.england.nhs.uk/statistics/statistical-work-areas/bed-availability-and-occupancy/.

[44] NHS. COVID-19 Hospital Activity. https://www.england.nhs.uk/statistics/statistical-work-areas/covid-19-hospital-activity/.

[45] Böhning D, Rocchetti I, Maruotti A, Holling H (2020). Estimating the undetected infections in the Covid-19 outbreak by harnessing capture–recapture methods. Int. J. Infect. Dis..

[46] Chen J (2020). Clinical progression of patients with COVID-19 in Shanghai, China. J. Infect..

[47] GNU General Public License, version 2.

[48] *Interactive UK Map*. https://wordpress.org/plugins/interactive-uk-map/.

[49] Garg, S. *et al.* Hospitalization Rates and Characteristics of Patients Hospitalized with Laboratory-Confirmed Coronavirus Disease 2019—COVID-NET, 14 States, March 1–30, 2020. *MMWR Morb. Mortal. Wkly. Rep.* **69**, (2020). 10.15585/mmwr.mm6915e3 PMC7755063 32298251

[50] Hong Y-R, Lawrence J, Williams D, Mainous A (2020). Population-level interest and telehealth capacity of US Hospitals in Response to COVID-19: cross-sectional analysis of google search and national hospital survey data. JMIR Public Health Surv..

