# Supplementary material for: A prognostic dynamic model applicable to infectious diseases providing easily visualized guides: a case study of COVID-19 in the UK
Source: Sci Rep. 2021 Apr 16;11:8412. doi: 10.1038/s41598-021-87882-9 (PMC8052322; doi:10.1038/s41598-021-87882-9)
Supplement: Supplementary file 1 — Supplementary information 1. [file 41598_2021_87882_MOESM1_ESM.docx]

**A** **prognostic dynamic model applicable to infectious diseases providing easily visualized guides —— A case study of COVID-19 in the UK**

Yuxuan Zhang^1,6†^, Chen Gong^2†^, Dawei Li^3†^, Zhi-Wei Wang^4,5^, Shengda D Pu^2^, Alex W Robertson^2^, Hong Yu^6^*, John Parrington^1^*

^1^ Department of Pharmacology, University of Oxford, Oxford OX1 3QT, United Kingdom.

^2^ Department of Materials, University of Oxford, Parks Road, Oxford OX1 3PH, United Kingdom.

^3^ Department of Physics, University of California, San Diego, La Jolla, CA, United States.

^4^ Computer Science, University of York, York YO10 5GH, United Kingdom.

^5^ College of Physics, Jilin University, Changchun 130012, People's Republic of China.

^6^ Shanghai Chest Hospital, Shanghai Jiao Tong University, Shanghai 200030, People's Republic of China.

^†^ Authors contributed equally to this work.

Correspondence to Prof. Hong Yu, Shanghai Chest Hospital affiliated to Shanghai Jiao Tong University [yuhongphd@163.com](mailto:yuhongphd@163.com), and Prof. John Parrington, Department of Pharmacology, University of Oxford. [john.parrington@pharm.ox.ac.uk](mailto:john.parrington@pharm.ox.ac.uk).

**Supplementary Materials**

**How to use the model and what kind of data you can get from the model**

Our prognostic dynamic model can be applied to any epidemic and is user-friendly. The input boxes include intraregional growth rate (m), interregional communication rate (c), percentage of hospitalization (h), and percentage of self-healing (s). To simulate the epidemic transmission process with disease control interventions, controlled intraregional growth rate (m_c_), controlled interregional communication rate (c_c_), detection rate of infectors (k), and the day of intervention are also required. The model can also be used in other regions beside the UK and London as long as the regional population (p) and population distribution are inputted and other parameters are changed according to the local conditions.

A video showing the transmission process on the regional map can be provided under each condition. The daily infection number is outputted together with the video and can also be outputted as an excel document. Cumulative infection number, daily hospitalization number (H), and daily self-healing number (S), can also be provided as an excel document based on the iterative algorithm.

**Detailed process of cellular automata establishment**

We set 9456 pixels and 16183 pixels, for the areas of London and the UK, respectively. Each pixel represents a 15 km^2^ geographic area, and people who travel over a 4 km (2.5 mile) straight-line distance are considered as migrants on the UK map (data collected from statistia.com), while on the London map, each pixel represents a 0.16 km^2^ geographic area, and people who travel over a pixel are regarded as migrants. As shown in Figure S1, there are four directions in which people in one cell can migrate, therefore, the number will be divided by 4. For example, if the travel parameter from one cell to another was estimated to be 0.1, this means that 10% people in one cell migrated between two adjacent cells every day, and 40% people in one cell migrated between cells.


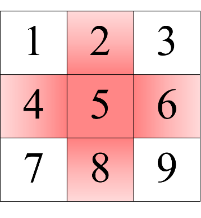


## Figure S1. Schematic diagram of automata cell establishment.

Each square cell in the diagram represents a pixel on the map. Cells 1-9 represent a region which can be a community, a village, a town or even a city. People tend to migrate between two adjacent cells, for example people in cell 5 can migrate to, or communicate with people in, cell 2, 4, 6, 8, and vice versa. Red saturation represents the severity of the epidemic. (r=0 means no cases, r=255 means the total population are infected).

**Timing of interventions is crucial for disease control**

We simulated the transmission process of COVID-19 in the UK with a combined intervention of intraregional growth rate (m_c_)=0.23, controlled interregional communication rate (c_c_)=0.08, and detection rate of infectors (k)=0.1, starting from day 16 and day 32 (Figure S2). From the infection curves we can clearly see that early intervention will significantly reduce the infection scale within 100 days while late intervention will lead to an out-of-control situation.


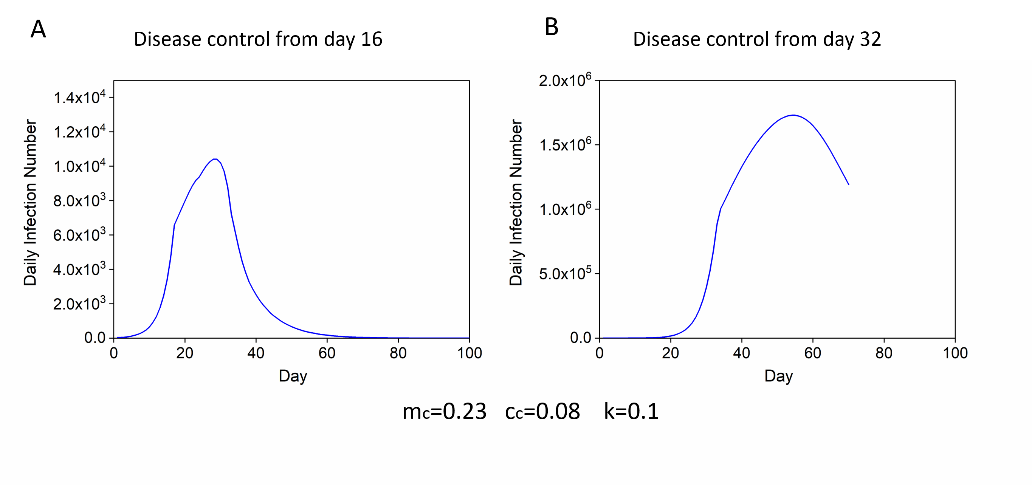


## Figure S2. The daily infection curves with the same control strategies starting from different time points.

(A/B) Daily infection curves in the UK with combined interventions of intraregional growth rate (m_c_)=0.23, controlled interregional communication rate (c_c_)=0.08, and detection rate of infectors (k)=0.1, starting from (A) day 16 / (B) day 32.

**Model verification**

Taking the progression of infection in the UK as an example, we simulated the regional infection numbers starting from 4^th^ March. We compared the confirmed infection numbers on 20^th^ March, one day before the government took coercive precautionary measures, and the simulated infection number on day 16. Here we assume 20% of infectors were [asymptomatic](http://www.youdao.com/w/asymptomatic/#keyfrom=E2Ctranslation) or had mild symptoms who were not detected but had the same infectious capacity; the confirmed numbers (3771 people) and 80% simulated numbers (3802 people) are roughly consistent. The simulated highest daily existing infection number is 14,812,660 which is roughly consistent with previous conjectures that a quarter of the UK population could contract COVID-19 without any intervention. Data about the real daily infection number was collected from coronavirus.data.gov.uk.

## Table S1. Confirmed, simulated and 80% simulated number on day16.

|  | **Confirmed number** | **Simulated number** | **80% simulated number** |
| --- | --- | --- | --- |
| **Day 16** | 3771 | 4752 | 3802 |

**Video Files**

Here we present several representative videos as references for the national disease control strategies and vaccination programme discussed in the main text. More videos can be generated by the model when parameters are modified. All videos were created by pythons 3 (<https://www.python.org>) and are publicly available on GitHub (<https://github.com/daweiliucsd/Cov19-model>).

Video file S1— Simulated 100-day transmission process in the UK with no intervention.

Video file S2— Simulated 100-day transmission process in London with no intervention.

Video file S3— Simulated 100-day transmission process in the UK when m_c_=0.3, c_c_=0.07, and k=0.155.

Video file S4— Simulated 100-day transmission process in the UK when m_c_=0.23, c_c_=0.07, and k=0.115.

Video file S5— Simulated 100-day transmission process in the UK when m_c_=0.17, c_c_=0.06, and k=0.045.

Video file S6— Simulated 60-day transmission process in the UK after vaccination promotion when m_c_=0.3, c_c_=0.1, and k=0.2.

Video file S7— Simulated 60-day transmission process in the UK after vaccination promotion when m_c_=0.23, c_c_=0.1, and k=0.13.

Video file S8— Simulated 60-day transmission process in the UK after vaccination promotion when m_c_=0.17, c_c_=0.1, and k=0.09.

Video file S9— Simulated 60-day transmission process in the UK after vaccination promotion when m_c_=0.1, c_c_=0.1, and k=0.05.

Video file S10— Simulated 60-day transmission process in London after vaccination promotion when m_c_=0.3, c_c_=0.2, and k=0.15.

Video file S11— Simulated 60-day transmission process in London after vaccination promotion when m_c_=0.23, c_c_=0.2, and k=0.11.

Video file S12— Simulated 60-day transmission process in London after vaccination promotion when m_c_=0.17, c_c_=0.2, and k=0.08.

Video file S13— Simulated 60-day transmission process in London after vaccination promotion when m_c_=0.1, c_c_=0.2, and k=0.05.
